# Orphan nuclear receptor Nur77 Inhibits Oxidized LDL-induced differentiation of RAW264.7 murine macrophage cell line into dendritic like cells

**DOI:** 10.1186/s12865-014-0054-z

**Published:** 2014-11-29

**Authors:** Liu-hua Hu, Ying Yu, Shu-xuan Jin, Peng Nie, Zhao-hua Cai, Ming-li Cui, Shi-qun Sun, Hua Xiao, Qin Shao, Ling-hong Shen, Ben He

**Affiliations:** Department of Cardiology, Renji Hospital, Shanghai Jiaotong University School of Medicine, Shanghai, 200127 People’s Republic of China

**Keywords:** Nuclear receptor, Nur77, Oxidized LDL, Macrophage, Dendritic cell

## Abstract

**Background:**

Nur77 is an orphan nuclear receptor expressed in human atheroma. In vascular cells *in vitro*, Nur77 expression is induced by pro-inflammatory factors, such as oxidized LDL (oxLDL).

**Methods:**

We analyze the role of Nur77 in the oxLDL-induced differentiation of macrophages into dendritic cells (DC). The murine RAW264.7 macrophage cell line was stably transfected with expression plasmids encoding either GFP or GFP fusions with either full-length Nur77 (GFP-Nur77), Nur77 lacking the DNA binding domain (GFP-Nur77-ΔDBD) or Nur77 lacking the transactivation domain (GFP-Nur77-ΔTAD).

**Results:**

GFP-Nur77 overexpression significantly suppressed the effect of oxLDL treatment on DC morphologic changes, expression of DC maturation markers, endocytic activity, allogeneic activation of T cell proliferation, and the activity and secretion of pro-inflammatory cytokines. Analysis of GFP-Nur77-ΔTAD and GFP-Nur77-ΔDBD indicated that the Nur77 DNA binding and transactivation domains were both required for this effect. GFP-Nur77-ΔDBD consistently had the opposite effect to GFP-Nur77, increasing DC-type differentiation in all assays. Interestingly, GFP-Nur77-ΔDBD protein was cytosolic, whereas GFP-Nur77 and GFP-Nur77-ΔTAD were both nuclear.

**Conclusions:**

These data show that GFP-Nur77 inhibited differentiation of oxLDL-treated macrophages into DC. The effects of Nur77 on the macrophage phenotype may involve changes in its subcellular distribution.

**Electronic supplementary material:**

The online version of this article (doi:10.1186/s12865-014-0054-z) contains supplementary material, which is available to authorized users.

## Background

Atherosclerosis is a chronic inflammatory disease of the arterial wall. From the first appearance of fatty streaks through to the development of advanced atheromatous plaques, atherosclerosis involves interactions between the immune system and metabolic risk factors. The immune and inflammatory actions of monocytes/macrophages play an important role in this disease [1-3]. Peripheral blood monocytes enter the arterial tunica intima, where they differentiate into macrophages or immature dendritic cells (DC) that differentiate fully after exposure to environmental factors [4]. We have shown *in vitro* that oxidized low-density lipoprotein (oxLDL) stimulates mature macrophages to differentiate into DC [5], a process that might be important for inflammatory and immune responses within the atheroma. Although there is currently no in vivo evidence for macrophage–DC differentiation, the functional consequences of this event might significantly exacerbate inflammation in the atheroma. The molecular mechanisms underlying macrophage–DC differentiation is essential for our understanding of atherogenesis and the development of novel drug therapies.

The orphan nuclear receptor Nur77, also known as NR4A1, NGFI-B or TR3, is a member of the steroid/thyroid hormone nuclear receptor superfamily, and was first identified as an early response gene to growth factor stimulation. These nuclear receptors are all transcription factors characterized by three main functional domains: a DNA-binding domain (DBD), which is flanked by an amino-terminal transactivation domain, and a carboxy-terminal hetero-dimerization and ligand binding domain. Nur77 is an orphan receptor since no specific ligands have been identified. Previous studies have identified diverse roles for Nur77 in cell proliferation, differentiation and apoptosis, as well as neuroendocrine regulation, neurological disorders, liver regeneration and cancer. Nur77 is expressed in human atherosclerotic lesions, and can be induced in human smooth muscle cells, macrophages and endothelial cells [6,7]. Pei LM et al. [8] found that many inflammatory stimuli, including oxLDL, elevate expression of Nur77 in macrophages in vitro, and we have found that Nur77 is upregulated in macrophages exposed to oxLDL [5].

Here, we have used *in vitro* approaches to investigate a possible role for Nur77 in oxLDL-induced macrophage–DC differentiation. We show that overexpression of Nur77 significantly inhibited the differentiation into DC of the RAW264.7 macrophage cells exposed to oxLDL. Analysis of deletion mutants of Nur77 indicated that the Nur77 DNA binding and transactivation domains were both required for this suppressive effect.

## Results

### Establishment of stable RAW264.7 cell lines expressing GFP-Nur77 and GFP-Nur77 deletion mutants

**Figure 1:**
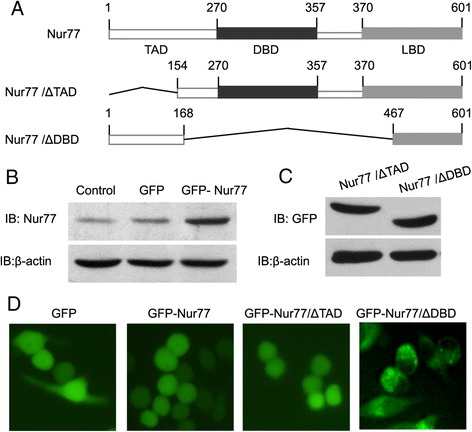
**Characterization of stable RAW264.7 cell lines expressing Nur77 and Nur77 deletion mutants. (A)** Schematic structure of the Nur77 gene and deletion mutants lacking either the transactivation domain (TAD) or DNA binding domain (DBD). **(B)** Expression of Nur77 protein in untransfected RAW264.7 cells and in RAW264.7 cell lines expressing GFP or GFP-Nur77. Expression of GFP-Nur77 is 3–4-fold higher than endogenous Nur77 in untransfected or GFP-transfected cells. **(C)** Expression of GFP-Nur77-ΔTAD and GFP-Nur77-ΔDBD fusion proteins in stably transfected RAW264.7 cells. β-actin expression was used to control for protein loading. A representative of three separate experiments is shown. **(D)** Subcellular localization of GFP-Nur77, GFP-Nur77-ΔTAD, and GFP-Nur77-ΔDBD in RAW264.7 cells.

We have shown previously that macrophages exposed to oxLDL in vitro differentiate into mature DC. Here, we have investigated a possible role for the orphan nuclear receptor Nur77 on the differentiation of oxLDL-treated RAW264.7 cells, a murine macrophage cell line. Nur77, a steroid/thyroid hormone nuclear receptor superfamily, contains three characteristic functional domains involved in transactivation, DNA binding, and ligand binding (Figure 1A). We established clonal RAW264.7 cell lines stably expressing either wild-type GFP-Nur77 or GFP fusion proteins with Nur77 lacking either the transactivation or DNA binding domains (GFP-Nur77-ΔTAD and GFP-Nur77-ΔDBD, respectively). GFP-Nur77 expression was 3–4 fold the level of endogenous Nur77 (Figure 1B). The two deletion mutants of Nur77 were expressed to similar extents (Figure 1C). Fluorescent microscopy revealed that GFP-Nur77-ΔDBD was cytosolic, whereas GFP-Nur77 and GFP-Nur77-ΔTAD were strictly nuclear (Figure 1D) suggesting that DNA binding is required for nuclear localization.

### Nur77 inhibits the differentiation of oxLDL-treated RAW264.7 cells

**Figure 2:**
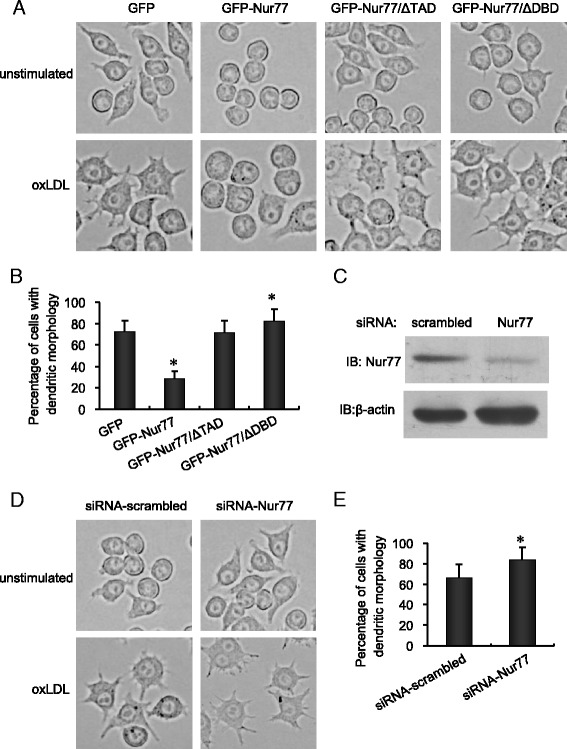
**Nur77 inhibits DC morphological changes in oxLDL**-**treated RAW264.7 cells. (A)** RAW264.7 cells stably expressing GFP-Nur77, GFP-Nur77-ΔTAD, or GFP-Nur77-ΔDBD were treated with oxLDL (10 μg/ml) for 48 h and visualized by phase contrast microscopy (400×). Results are representative of three separate experiments. **(B)** Cells with DC morphology were calculated as the percentage of all cells observed in 10 different fields at 400× magnification. The bars represent mean ± SD from three experiments. *p <0.05 compared with GFP-expressing control. **(C)** Western blots showing endogenous Nur77 in RAW264.7 cells 48 h after transfection with either scrambled siRNA or Nur77 siRNA. Similar results were obtained in three separate experiments. **(D)** Phase contrast images showing the morphology of oxLDL-treated RAW264.7 cells transfected with either scramble siRNA or Nur77 siRNA. Cultured cells were visualized by phase contrast microscopy as described in **(A)** and cells with DC morphology were calculated as described in **(B)**. **(E)** *p <0.05 compared with scrambled control.

We tested the effects of oxLDL on the morphology, DC surface marker expression, endocytic activity, allostimulatory activity, and cytokine secretion of the RAW264.7 stable cell lines. Consistent with previous results, 72.50% of GFP control cells had DC morphology after oxLDL treatment as determined by increased cell size, the presence of multiple prominent cytoplasmic processes, and prominent nucleoli (Figure 2A and B). In contrast, although most GFP-Nur77-expressing cells increased in size, only 28.94% had DC morphology following oxLDL treatment. In contrast, 72.30% oxLDL-treated GFP-Nur77-ΔTAD or 82.8% of oxLDL-treated GFP-Nur77-ΔDBD cell lines were of DC morphology, which was similar to control GFP-expressing cells (*p* >0.05). There was a small but statistically significant increase in the proportion of DCs in GFP-Nur77-ΔDBD cells compared to GFP-expressing cells (*p* <0.05; Figure 2A,B). To determine whether endogenous Nur77 played a role in macrophage–DC differentiation, we used siRNAs to deplete Nur77 and assayed the effect on oxLDL-induced morphological changes. Transfection of *Nur77* siRNA successfully depleted endogenous Nur77 in RAW264.7 cells compared to the scrambled siRNA (Figure 2C) and led to a 17% increase in the proportion of cells with DC morphology following oxLDL treatment compared to that in the scrambled siRNA group ( 66.5 ± 12.4% *vs*. 83.8 ± 12.1%, *p* <0.05; Figure 2D,E).

To provide definitive evidence to the observed morphology, CD209 was tested by confocal microscopy. (Detailed descriptions of the materials and experimental methods are available in Additional files 1 and 2).

### Nur77 inhibits phenotypic changes in oxLDL-treated RAW264.7 cells

**Figure 3:**
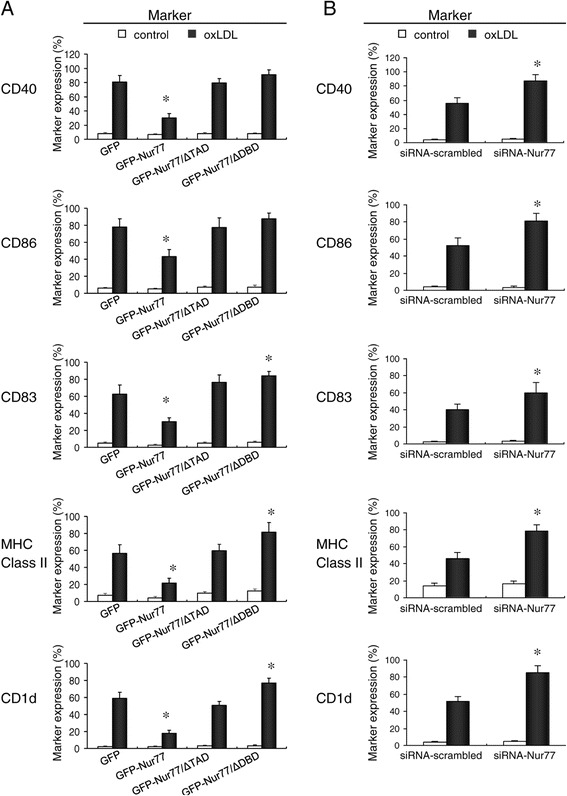
**Flow cytometry analysis of the cell surface phenotype of oxLDL**-**treated RAW264.7 cell lines. (A)** Expression of CD40, CD86, CD83, MHC Class II, and CD1d in RAW264.7 cells lines treated with oxLDL for 48 h. Mean ± SD of three independent experiments is shown. *p <0.05 compared with GFP-expressing control. **(B)** Expression of CD40, CD86, CD83, MHC Class II, and CD1d in RAW264.7 cells transfected with either scrambled siRNA or Nur77 siRNA and stimulated with oxLDL for 48 h. *p <0.05 compared with scrambled control.

The changes in cell morphology described above suggest that Nur77 inhibits oxLDL-induced RAW264.7 cell differentiation into DCs through its DNA binding and transactivation domains. To investigate this possibility, we evaluated phenotypic changes in oxLDL-treated RAW264.7 cells stably expressing Nur77 and Nur77 mutant proteins by flow cytometry using specific antibodies against co-stimulatory molecules, antigen-presenting molecules, and markers of DC activation. Following oxLDL treatment, the levels of CD40, CD86, CD83, MHC class II, and CD1d were reduced by 62.4%, 44.69%, 51.7%, 55.2%, and 53.29%, respectively, in RAW264.7 cells stably expressing GFP-Nur77 protein compared with those in GFP-expressing cells. However, there was little difference in the levels of these proteins when comparing cells expressing either GFP-Nur77-ΔTAD or GFP-Nur77-ΔDBD with GFP-expressing cells, with the exception that GFP-Nur77-ΔDBD cells had a small but significant increase in the expression of CD83, MHC class II molecules, and CD1d (*p* <0.05 compared to control cells; Figure 3A). Moreover, all of the aforementioned proteins were significantly up-regulated in Nur77-depleted RAW264.7 cells when compared to control cells transfected with scrambled siRNA (*p* <0.05; Figure 3B).

### Nur77 decreased endocytosis in oxLDL-treated RAW264.7 cells

**Figure 4:**
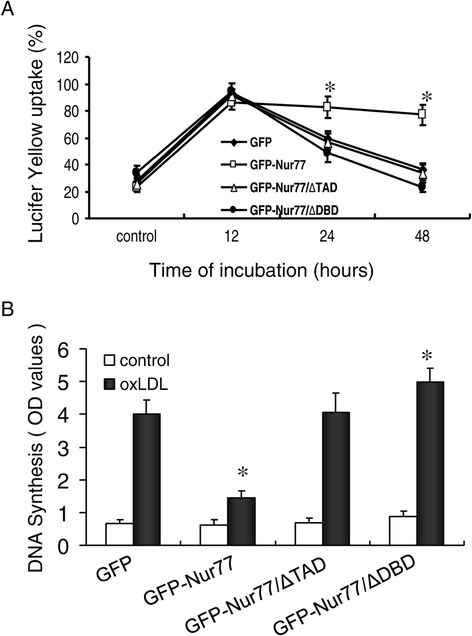
**Nur77 enhanced the endocytic activity but suppressed the DNA synthesis of RAW264.7 treated by oxLDL. (A)** Flow cytometry analysis of endocytic activity using lucifer yellow (LY) uptake by oxLDL-treated RAW264.7 cells stably expressing GFP-Nur77, GFP-Nur77-ΔTAD, or GFP-Nur77-ΔDBD. Mean ± SD of three independent experiments is shown. *p <0.05 compared with GFP-expressing control. **(B)** Flow cytometry analysis of BrdU incorporation by co-cultures of allogeneic T cells and RAW264.7 cells stably expressing either GFP, GFP-Nur77, GFP-Nur77-ΔTAD, or GFP-Nur77-ΔDBD. Mean ± SD of three independent experiments is shown. *p <0.05 compared with GFP-expressing control.

We have previously shown that oxLDL-treated RAW264.7 cells gain several of the functional characteristics of mature DC [5]. Therefore, we next assayed the role of Nur77 in these oxLDL-induced functional changes. Using LY uptake as a marker of endocytosis (Figure 4A), we found that a large proportion of the four stable RAW264.7 cell lines were LY + following 12 h exposure to oxLDL. Consistent with published results, oxLDL had a dynamic effect on endocytosis. GFP-expressing controlcells exhibited decreased LY uptake by approximately 33% at 24 h and 57% at 48 h after oxLDL treatment. Similar results were obtained from GFP-Nur77-ΔTAD and GFP-Nur77-ΔDBD-expressing cells, except that at 48 h LY uptake by GFP-Nur77-ΔDBD-expressing cells was 15% lower than GFP-expressing cells. In contrast, oxLDL had little effect on LY uptake in GFP-Nur77-expressing cells at either 24 h or 48 h. These results indicate that overexpression of Nur77 protein in RAW264.7 cells inhibited the endocytic changes typically displayed by mature DCs, and that this inhibition was dependent upon the DNA binding and transactivation domains of Nur77.

### Nur77 reduced the stimulatory effect of oxLDL-treated RAW264.7 cells on T cell proliferation

Stimulation of allogeneic T cell proliferation is an important function of mature DC. Therefore, we evaluated the effect of Nur77 on T cell proliferation. RAW264.7 cells stably expressing either Nur77 or the Nur77 mutant proteins were pretreated with 10 μg/ml oxLDL for 48 hrs and then cultured with T cells at a ratio of 1:10 for 5 days. To detect T cell proliferation, we monitored BrdU incorporation as a measure of cells entering S phase. In the absence of oxLDL, there was no T cell proliferation in any of the four stable cell lines. OxLDL-treated GFP-expressing control cells effectively induced the proliferation of T cells, as did GFP-Nur77-ΔTAD-expressing and GFP-Nur77-ΔDBD-expressing cells. In contrast, T cell proliferation in co-cultures with GFP-Nur77-expressing cells was approximately one third of the other cell lines (Figure 4B). These results are consistent with an inhibitory role for Nur77 in this T cell assay, possibly through reduced expression of co-stimulatory molecules.

### Nur77 reduced cytokine secretion by oxLDL-treated RAW264.7 cells

**Figure 5:**
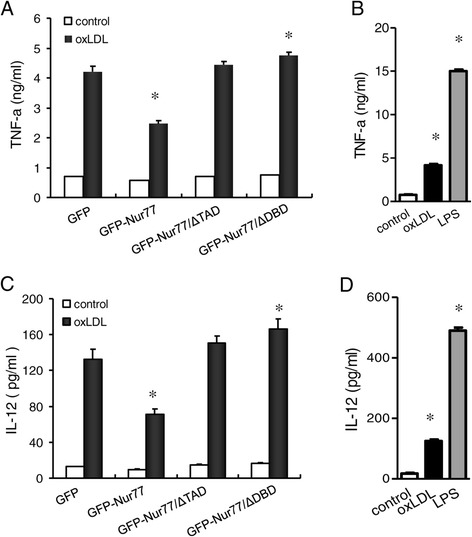
**Nur77 overexpression in RAW264.7 cells reduces oxLDL**-**induced inflammatory cytokine synthesis.** Sandwich ELISA analysis of TNF-α **(A)** and IL-12 **(C)** for the supernatants of oxLDL-treated RAW264.7 cells stably expressing GFP, GFP-Nur77, GFP-Nur77-ΔTAD or GFP-Nur77-ΔDBD. Data represent mean ± SD of three independent experiments for 1 × 10^6^ cells, *p <0.05 compared with GFP-expressing control cell. **(B)** and **(D)** showed the level of TNF-α and IL-12 in GFP-expressing control cells with or without stimulated by oxLDL or LPS, respectively. The bars represent mean ± SD from three experiments. *p <0.05 compared with control cells.

Finally, we assayed the effect of Nur77 on cytokine secretion by oxLDL-treated RAW264.7 cells. Here we show that, IL-12 and TNF-α from oxLDL-treated GFP-Nur77 cells were decreased to 46.8% and 41.5%, respectively, compared with that from GFP-expressing control cells. In contrast, expression of the Nur77 mutant proteins did not decrease oxLDL-induced IL-12 and TNF-α secretion. GFP-Nur77-ΔDBD-expressing cells demonstrated a 24.9% and 12.7% increase in IL-12 and TNF-α secretion, respectively (Figure 5A,C). These data show that Nur77 had an inhibitory effect on cytokine secretion, which required the Nur77 DNA binding domain and transactivation domains. To test the ability of producing several cytokines by these cells, we also analyzed TNF-α and IL-12 including a stimulated control with LPS in GFP-expressing control cells. Results showed that both of the two cytokines can be stimulated by LPS and the levels were 3-fold higher than by the stimulation with oxLDL (TNF-α: 490.12 ± 14.14 *vs*. 125.60 ± 7.07 ng/ml; IL-12: 15.47 ± 0.28 *vs*. 4.15 ± 0.21 pg/ml ) (Figure 5B,D).

## Discussion

Dendritic cells are the most potent of all antigen-presenting cells, and play a key role in vascular inflammation and immune activation [9]. OxLDL is a particularly potent inducer of inflammation during the development of atherosclerosis [10,11]. Here, we have shown that Nur77 is likely to play an inhibitory role in DC differentiation. We found that overexpression of Nur77 in a macrophage cell line, RAW264.7, prevented differentiation into mature DCs following oxLDL treatment, whereas siRNA-mediated Nur77 depletion had the opposite effect. Consistent with this, constitutive expression of Nur77 in vivo decreases immune cell development and maturation. Together, these data suggest that endogenous Nur77 may have an inhibitory effect on macrophage activation and immunity. Many studies of vascular cells also suggest a protective role for Nur77 in atherogenesis [12-14]. For example, overexpression of Nur77 in arterial smooth muscle reduces the formation of smooth muscle-rich lesions [13]. In addition, in vitro assays have demonstrated that Nur77 significantly reduces the inflammatory response and lipid-loading of human macrophages [15,16].

The possible role of Nur77 in endothelial cells remains unclear, however. In human vascular endothelial cells, Nur77 mediates cell cycle arrest through up-regulation of p27Kip1 and downregulation of cyclin A [17], whereas others have implicated Nur77 in vascular endothelial growth factor (VEGF)-A-induced angiogenesis [18,19]. Thus, the role of Nur77 in human endothelial cell proliferation is controversial. The reasons for these contradictory effects on endothelial cell proliferation are not clear, but similar effects of Nur77 are observed in other cells. For example, Nur77 was originally recognized for its role in cancer cell proliferation [20-22] but was later found to be a potent pro-apoptotic molecule [23,24]. Extensive evidence indicates that the stimulation of proliferation by Nur77 is a transcriptional effect, based on the observations that the DNA binding and transactivation domains were both required [25]. We have also found that the transcriptional activity of Nur77 is required for suppression of macrophage activation [16]. Thus, we were interested in whether the inhibitory effect of Nur77 on oxLDL-induced macrophage differentiation was also dependent upon its transcriptional activity. Consistent with this, expression of GFP-Nur77ΔTAD had no significant effect on the induction of DC differentiation by oxLDL compared with expression of GFP alone. In contrast, expression of GFP-Nur77-ΔDBD caused a modest but significant increase in the proportion of cells with DC morphology and functionality.

The subcellular distribution of the GFP-Nur77 fusion proteins is consistent with a transcriptional role for Nur77 because GFP-Nur77-ΔDBD was mainly cytosolic, whereas GFP-Nur77 and GFP-Nur77-ΔTAD were nuclear. These observations suggest that Nur77 protein may induce the differentiation of macrophages into DC when it is localized in the cytosol. Although it is not known if oxLDL induces the nuclear export of Nur77 in macrophages, there is evidence from cancer cells that Nur77 can be induced to translocate from the nucleus to the cytosol and that this confers important biological functions, including apoptosis and differentiation.

## Conclusion

the differentiation of macrophages into DC following exposure to oxLDL can be suppressed by the transcriptional activity of Nur77. The effect of Nur77 on macrophage differentiation may be related to changes in its subcellular location. Nur77 might induce macrophage-DC differentiation when it localized in the cytosol. Further studies are needed to understand the relationship between the subcellular location of Nur77 and its protective effect against atherosclerosis.

## Methods

### Lipoprotein isolation and oxidation

LDL (d = 1.019–1.063) was isolated by sequential ultracentrifugation of EDTA-anticoagulated fasting plasma from healthy normo-lipidemic volunteers. After ultracentrifugation, EDTA was dialyzed at 4°C against PBS containing 0.5 mM EDTA. The LDL protein concentration was determined by a modification of the Lowry method, using bovine albumin as the standard. Isolated LDL (1 mg/ml) was oxidized at 37°C for 18 h in PBS containing 5 μM CuSO_4_. Oxidation reactions were terminated by the addition of EDTA (0.3 mM, pH 8.5) and butylated hydroxytoluene (40 μM). Modified LDL was dialyzed extensively at 4°C against PBS containing 0.5 mM EDTA, and the oxLDL preparations were sterilized by membrane filtration (0.45 μm) and stored in the dark at 4°C. All lipoprotein preparations were tested for endotoxin using an endotoxin assay kit (Sigma, St. Louis, MO).

The study protocol were approved by the Ethics Committee of Renji Hospital, School of Medicine, Shanghai Jiao tong University and complied with the National Regulations on the Use of Clinical Samples in China. These volunteers provided written informed consent to Ethics Committee for research purpose.

### Cell culture

RAW264.7 cells were obtained from the American Type Culture Collection, and grown at 37°C, 5% CO_2_ in DMEM containing penicillin (100 U/ml), streptomycin (100 μg/ml), and 10% heat-inactivated fetal calf serum.

### Plasmid and transient transfection of RAW264.7 cells

The GFP-Nur77 expression plasmid (pGFP-Nur77), GFP-Nur77-ΔTAD and GFP-Nur77-ΔDBD were gifts from X-K. Zhang (Burnham Institute, La Jolla, CA). RAW264.7 cells were plated into 6-well plates, 1 × 10^6^ cells per well, incubated overnight, then transfected with Lipofectamine 2000 (Invitrogen, USA) and 7.5 μg DNA per well, according to the manufacturer’s instructions.

### Establishment of stable clones expressing GFP-Nur77, GFP-Nur77-ΔTAD, and GFP-Nur77-ΔDBD

*pGFP*-*Nur77*, *pGFP*-*Nur77*-*ΔTAD*, *pGFP*-*Nur77*-*ΔDBD*, and the *pGFP*-*control* vectors were introduced into RAW264.7 cells as described [5]. Transfected cells were allowed to recover for 24 h, followed by drug selection using 500 μg/ml G418 for a week. Single cell clones were identified by serial dilution in 96 well plates. Clonal RAW264.7 cell lines stably transfected with pGFP-Nur77, pGFP-Nur77-ΔTAD, pGFP-Nur77-ΔDBD or *pGFP* vectors were incubated in medium containing 200 μg/ml G418. Overexpression of GFP-Nur77, GFP-Nur77-ΔTAD and GFP-Nur77-ΔDBD were confirmed by fluorescent microscopy and western blot analysis.

### siRNA transfections

The following small interfering RNAs (siRNAs; Dharmacon Research Inc.) were used: Nur77, 5’-CAG UCC AGC CAU GCU CCU C dTdT-3’; scrambled siRNA, 5’-GCG CGC TTT GTA GGA TTC G dTdT-3’. siRNAs were transfected (10-μl aliquot of 20 μM siRNA/well) into individual wells of 6-well plates using Oligofectamine (Invitrogen), according to the manufacturer’s protocol. Two days after transfection, cells were harvested for analysis.

### Western blotting

Cells were lysed in 150 mM NaCl, 10 mM Tris (pH 7.5), 5 mM EDTA, 1% Triton X-100, 1 mM PMSF, 10 mg/ml leupeptin, 10 mg/ml pepstatin, and 10 mg/ml aprotinin for 30 min on ice. Equal amounts of lysates (50 μg) were separated by 8–12% SDS-PAGE and transferred onto Immobilon-P transfer membranes (Millipore, Billerica, MA). Non-specific binding sites were blocked by incubating membranes in 5% (w/v) solution of nonfat dried milk in TBST (50 mM Tris–HCl (pH 7.4), 150 mM NaCl, 0.1% Tween 20). Blocked membranes were probed with specific antibodies against Nur77 (1;1000, Abcam, Cambridge, UK), GFP (1;1000, eBioscience, San Diego, CA), or β-actin (1;5000, BioVision, Mountain View, CA) in TBST. Membranes were washed 3 times with TBST and then incubated for 2 h at room temperature in TBST containing HRP-linked anti-rabbit or anti-mouse immunoglobulin. After 3 washes in TBST, HRP was visualized by chemiluminescence using the ECL kit (Chemicon, Temecula, CA). The protein concentration was quantified using the BCA protein assay (Merck, Darmstadt, Germany).

### Phenotypic analysis by flow cytometry

Cells were incubated for 30 min with Phycoerythrin (PE)-labeled monoclonal antibodies recognizing either CD40, CD83, CD86, MHC Class II, or CD1d (eBioscience), washed with staining buffer, and analyzed by flow cytometry on a FACSCalibur (BD Biosciences, Franklin Lakes, NJ).

### Analysis of endocytic activity

Endocytosis was measured after treatment with oxLDL (10 μg/ml) for the indicated times. Cells were incubated at 37°C for 30 min with Lucifer Yellow (LY; 1 mg/ml; Sigma-Aldrich), and washed 3 times at 4°C with cold PBS containing 1% BSA and 0.05% NaN3. Cells were analyzed on a FACSCalibur (BD Biosciences).

### T cell proliferation assay

The effect on allogeneic T cell proliferation of oxLDL-treated RAW264.7 cells was measured as described [13]. Briefly, T cells were purified from the spleens of C57BL/6 mice using nylon wool columns. RAW264.7 cells grown in 96-well tissue culture plates were treated with oxLDL for 48 h and then treated with mitomycin C (50 μg/ml) for 1 h. Afterwards, a total of 1 × 10^5^ T cells were added as responders. The cell mixtures were cultured for 5 days and 20 μl diluted BrdU was added 18 h prior to the end of the incubation period according to the procedure recommended by the manufacturer (Chemicon). The plates were analyzed using a spectrophotometer microplate reader with the absorbance set at 450 nm (MK3, Thermo, USA).

### Cytokine assays

GFP-expressing RAW264.7 cells were stimulated by LPS (5 μg/ml, Sigma) or oxLDL (10 μg/ml) for 24 h, and secretion of IL-12 or TNF-α into the supernatant was analyzed using cytokine specific ELISA kits according to manufacturer instructions (BD Bioscience).

### Statistical analysis

Data were expressed as mean ± SD. One way-ANOVA. When more than two groups were involved in analysis, Dunnett test was used for comparison of difference between two groups. Comparisons with p <0.05 were considered statistically significant. Comparisons with p <0.05 were considered statistically significant.

## Additional files

Additional file 1:
**Supplemental materials and methods.**
Additional file 2: Figure S1. Nur77 inhibits DC-specific marker CD209 changes in oxLDL-treated RAW264.7 cells. **(A)** RAW264.7 cells stably expressing GFP, GFP-Nur77, GFP-Nur77-ΔTAD or GFP-Nur77-ΔDBD were treated with oxLDL (10 μg/ml) for 48 h and visualized by fluorescent microscopy (200×). Results are representative of three separate experiments. **(B)** Cells with CD209 positive staining were calculated as the percentage of all cells observed in 10 different fields at 200× magnification. The bars represent mean ± SD from three experiments. **p* <0.05 compared with GFP-expressing control cells.

## Abbreviations

oxLDL: Oxidized LDL
DC: Dendritic cells
DBD: DNA-binding domain
GFP-Nur77: GFP fusions with either full-length Nur77
GFP-Nur77-ΔDBD: Nur77 lacking the DNA binding domain
GFP-Nur77-ΔTAD: Nur77 lacking the transactivation domain
VEGF: Vascular endothelial growth factor
