# Supplementary material for: Orphan nuclear receptor Nur77 Inhibits Oxidized LDL-induced differentiation of RAW264.7 murine macrophage cell line into dendritic like cells
Source: BMC Immunol. 2014 Nov 29;15:54. doi: 10.1186/s12865-014-0054-z (PMC4274730; doi:10.1186/s12865-014-0054-z)
Supplement: Additional file 1: — Supplemental materials and methods. [file 12865_2014_54_MOESM1_ESM.doc]

**Supplemental data**

**Methods：**

1．Immunofluorescen

1×10^5^ cells were seeded on slides in 12-well plant, after stimulation with oxLDL, blocked with 5% FCS for 30 minutes and incubated with primary antibodies against CD209 (sc-241347, dilution 1:100, santa cruz) overnight. Secondary antibody marked with red fluorescence (donkey-anti-mouse, 555 nm) was used to visualize the primary antibodies. After 60-minute incubation, the slides were observed by fluorescent microscopy and analyzed using image analysis software (Image J). The number of positive cells was evaluated as percentage of 100 cells in 10 independent fields of visions.

**Results：**

1．Nur77 inhibits DC-specific marker CD209 in oxLDL-treated RAW264.7 cells

Following oxLDL treatment, the expression of DC-specific antibody, CD209, was detected by fluorescent microscopy. We found that, consistent with the trend in phenotype changes, CD209 was reduced by nearly 25% in RAW264.7 cells stably expressing GFP-Nur77 (40.28±2.28%) protein compared with those in GFP-expressing control cells (65.33±3.54%). Meanwhile, there was significant difference in the level of CD209 when comparing cells expressing GFP-Nur77-ΔDBD (74.33±2.64%) with GFP-expressing control cells, but no statistical difference existed between cells expressing GFP-Nur77-ΔTAD (65.01±2.58%) and the control cells.

2. Nur77 reduced cytokine secretion by oxLDL-treated RAW264.7 cells

To test the ability of producing several cytokines by these cells, we analyzed TNF-α and IL-12 including a stimulated control with LPS in GFP-expressing control cells. Results showed that both of the two cytokines can be stimulated by LPS and the levels were 3-fold higher than by the stimulation with oxLDL (TNF-α: 490.12±14.14 *vs.* 125.60±7.07 ng/ml; IL-12: 15.47±0.28 *vs.* 4.15±0.21pg/ml ).
