# Supplementary figures and images for: Orphan nuclear receptor Nur77 Inhibits Oxidized LDL-induced differentiation of RAW264.7 murine macrophage cell line into dendritic like cells
Source: BMC Immunol. 2014 Nov 29;15:54. doi: 10.1186/s12865-014-0054-z (PMC4274730; doi:10.1186/s12865-014-0054-z)

## Slide 1
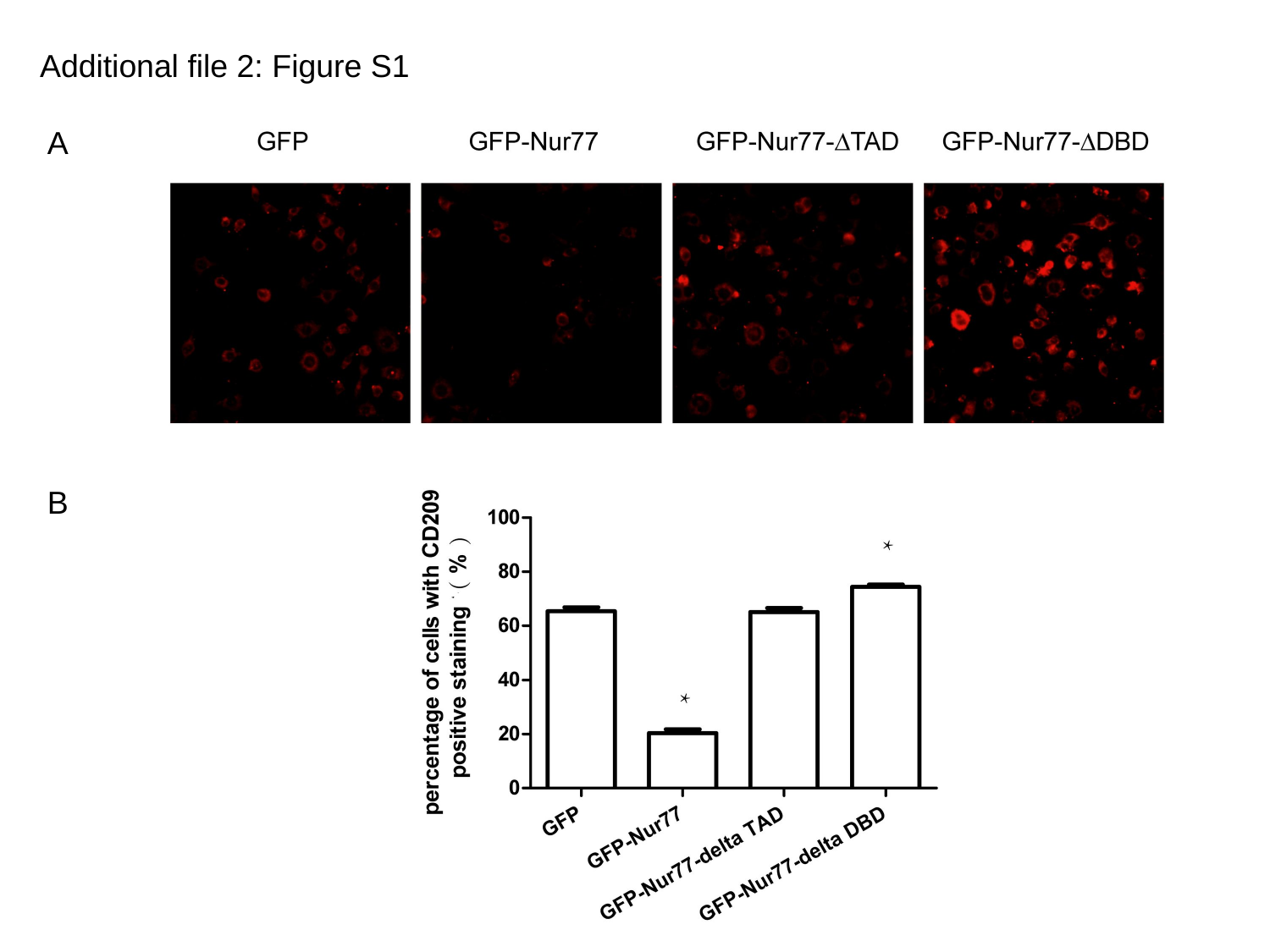

Additional file 2: Figure S1
A
B

Supplement: Additional file 2: Figure S1. — Nur77 inhibits DC-specific marker CD209 changes in oxLDL-treated RAW264.7 cells. (A) RAW264.7 cells stably expressing GFP, GFP-Nur77, GFP-Nur77-ΔTAD or GFP-Nur77-ΔDBD were treated with oxLDL (10 μg/ml) for 48 h and visualized by fluorescent microscopy (200×). Results are representative of three separate experiments. (B) Cells with CD209 positive staining were calculated as the percentage of all cells observed in 10 different fields at 200× magnification. The bars represent mean ± SD from three experiments. *p <0.05 compared with GFP-expressing control cells. [file 12865_2014_54_MOESM2_ESM.ppt]
